# Exploratory assessment of cerebrospinal fluid-related microdynamics after mild traumatic brain injury using intravoxel incoherent motion magnetic resonance imaging

**DOI:** 10.3389/fnins.2026.1756207

**Published:** 2026-04-10

**Authors:** Shinya Watanabe, Yasushi Shibata, Eiichi Ishikawa

**Affiliations:** 1 Department of Neurosurgery, Mito Kyodo General Hospital, Tsukuba University Hospital Mito Area Medical Education Center, Mito, Japan; 2 Institute of Medicine, University of Tsukuba, Tsukuba, Japan

**Keywords:** cerebrospinal fluid, glymphatic system, intravoxel incoherent motion, magnetic resonance imaging, neurofluid dynamics, traumatic brain injury

## Abstract

**Aim:**

This study aimed to characterize regional alterations in cerebrospinal fluid (CSF) microdynamics following mild traumatic brain injury (TBI) using intravoxel incoherent motion (IVIM) magnetic resonance imaging (MRI) and compare *f*-value–based CSF motion between patients with TBI and healthy controls.

**Methods:**

In this prospective observational study, 14 patients with mild TBI and 14 healthy volunteers underwent IVIM MRI using a 3-Tesla scanner. The *f*-value, reflecting incoherent CSF-related microfluidic motion, was quantified across predefined supratentorial and infratentorial regions of interest. Group differences in mean *f*-values were evaluated, and longitudinal changes were assessed in three patients who underwent follow-up MRI.

**Results:**

The TBI group exhibited a significantly higher mean *f*-value in the left cerebellopontine angle (CPA) compared with controls. Exploratory trends toward lower *f*-values were also observed in several supratentorial regions, including the left lateral ventricle and right frontal subarachnoid space. Longitudinal analysis revealed increasing *f*-values in supratentorial regions over time—suggesting partial recovery—whereas infratentorial regions demonstrated decreasing or stable trajectories.

**Conclusion:**

Mild TBI may be associated with region-specific alterations in CSF microdynamics, characterized by increased motion in the CPA and exploratory reductions in selected supratentorial regions. Although preliminary, these findings highlight the potential of IVIM MRI as a complementary tool for investigating post-traumatic abnormalities in CSF motion.

## 1 Introduction

Understanding of cerebrospinal fluid (CSF) and interstitial fluid (ISF) dynamics has advanced rapidly over the past decade. Major discoveries—including the glymphatic system described by Nedergaard (2013), the meningeal lymphatic vessels identified by Louveau et al. (2015), intramural perivascular drainage clarified by Engelhardt et al. (2017), and the subarachnoid lymphatic-like membrane recently reported by Møllgård et al. (2023)—have reshaped current concepts of neurofluid circulation. These interconnected pathways are essential for metabolic waste clearance and immune regulation, and dysfunction within these systems has been implicated in Alzheimer’s disease, cerebral amyloid angiopathy, and idiopathic normal pressure hydrocephalus (Kwon et al., 2019; Miura et al., 2022; Ullah and Lee, 2023). Despite these advances, the effects of traumatic brain injury (TBI) on CSF/ISF dynamics remain incompletely understood. Post-traumatic conditions such as subdural effusion, chronic subdural hematoma, and secondary hydrocephalus frequently emerge during the subacute phase (Lindfors et al., 2023), and chronic complications—including post-traumatic Alzheimer’s disease and Parkinson’s syndrome—have also been reported (Daniela et al., 2023; Kitoka et al., 2024; Moody et al., 2024; Wee et al., 2024). Although glymphatic and related clearance pathways may be disrupted following injury, the association between mild TBI and neurofluid dysfunction has only recently gained attention (Miettinen et al., 2025). The underlying mechanisms remain unclear; however, accumulating evidence suggests that alterations in CSF/ISF motion may contribute to post-traumatic pathophysiology.

Recent advances in diffusion magnetic resonance imaging (MRI) have enabled indirect evaluation of CSF dynamics through established and emerging techniques (Wright et al., 2024). Among these approaches, intravoxel incoherent motion (IVIM) MRI has been applied to various neurological conditions, including for the identification of patients with moyamoya disease at risk of postoperative hyperperfusion syndrome (Gao et al., 2022), the characterization of idiopathic intracranial hypertension (Watanabe et al., 2024), and the assessment of incoherent CSF motion along cortical surfaces (Yamada et al., 2023). Animal studies have further demonstrated glymphatic inflow impairment following TBI (Plog and Nedergaard, 2018). IVIM MRI separates diffusion and incoherent motion components of water molecules and has been used to probe microvascular or microdynamic fluid motion without contrast agents. Therefore, IVIM MRI is particularly well suited for exploring post-traumatic neurofluid alterations, as its *f*-value reflects microperfusion and incoherent fluid motion, enabling regional assessment across both supratentorial and infratentorial compartments. However, despite increasing interest in glymphatic dysfunction after TBI, no study has systematically examined regional CSF microdynamics using IVIM MRI in humans. Therefore, the present study aimed to investigate regional alterations in CSF motion in patients with mild TBI using IVIM MRI. Specifically, the study compared the *f*-value–based CSF microdynamics between patients with mild TBI and healthy individuals to characterize acute-phase post-traumatic changes.

## 2 Methods

### 2.1 Study design

This prospective observational study assessed CSF motion in patients with mild TBI using IVIM MRI. The study was performed in accordance with the Strengthening the Reporting of Observational Studies in Epidemiology guidelines (von Elm et al., 2007) and adhered to the principles outlined in the Declaration of Helsinki. The study was approved by the Institutional Review Board of Mito Kyodo General Hospital (approval no. NO23-09). Written informed consent was obtained from all participants.

### 2.2 Participants

**Figure 1:**
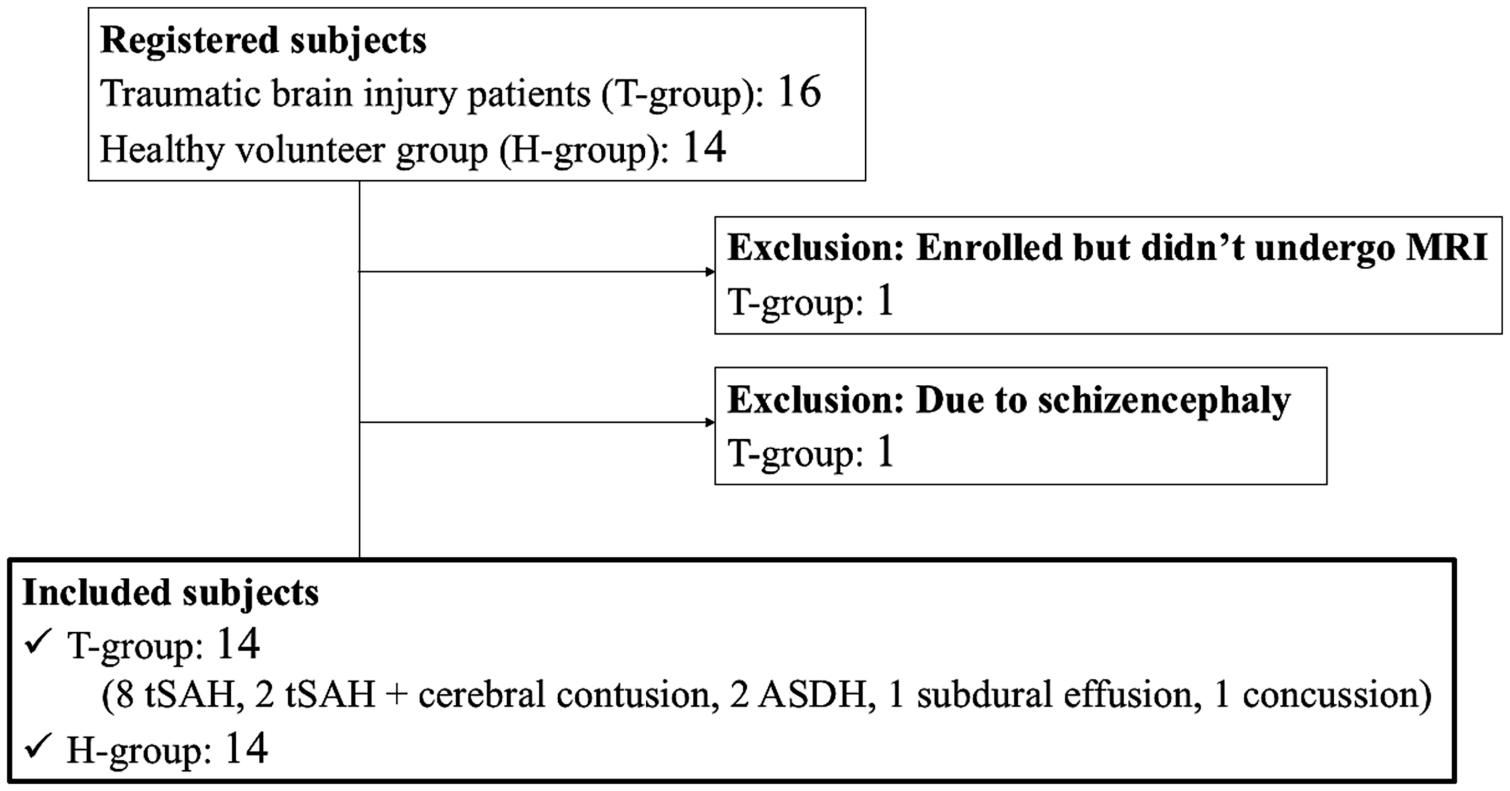
Flowchart illustrating the study design and participant selection process. MRI, magnetic resonance imaging; tSAH, traumatic subarachnoid hemorrhage; ASDH, acute subdural hematoma.

The study participants with mild TBI presented with traumatic subarachnoid hemorrhage (tSAH), acute subdural hematoma (ASDH), and brain contusion. Patients who sustained a minor head injury with a Glasgow Coma Scale (Teasdale and Jennett, 1974) score of ≥13 points were eligible for study inclusion, whereas individuals aged <18 years were excluded. Initially, 16 patients with trauma and 14 healthy volunteers were enrolled in this study. One patient with TBI who was unable to undergo MRI and another with schizencephaly were excluded. Consequently, only 14 patients with TBI (T-group) and 14 healthy volunteers (H-group) were included in the final analysis. The T-group comprised eight patients with tSAH, including two with cerebral contusion, two with ASDH, one with subdural effusion, and one with concussion (Figure 1). The causes of injury in this group were primarily falls (*n* = 11), traffic accidents (*n* = 2), and work-related injuries (*n* = 1).

### 2.3 MRI acquisition protocol

All MRI scans were acquired using a 3-Tesla MRI scanner (MAGNETOM Skyra, Siemens Healthineers, Erlangen, Germany) equipped with a 20-channel head coil. The imaging protocol included diffusion-weighted imaging (DWI) sequences optimized for IVIM analysis. The repetition time was set to 5,700 ms, whereas the echo time was set to 64 ms. The acquisition matrix was 156 × 156 (interpolated to 312 × 312), with a reconstructed voxel size of 0.71 × 0.71 × 4.0 mm^3^ and a field of view of 220 × 220 mm^2^. DWI was performed using 12 *b*-values of 10, 20, 30, 40, 80, 100, 200, 250, 400, 500, 800, and 1,000 s/mm^2^ in three orthogonal diffusion directions. Direction-averaged images were used for IVIM fitting. Parallel imaging was implemented using the GRAPPA technique (acceleration factor PE: 3), resulting in a total acquisition time of approximately 4 min. Image reconstruction was performed using the vendor’s standard pipeline, which included sensitivity normalization (Normalize) and a raw uniformity filter to improve signal uniformity across the field of view. No dedicated denoising or additional signal-to-noise ratio (SNR)-enhancing post-processing was applied. Respiratory motion artifacts were minimal because imaging was performed in the brain; therefore, respiratory gating was not applied. In addition, IVIM MRI reflects incoherent microdynamic motion within a voxel rather than time-resolved bulk flow.

IVIM parametric maps (*f*, *D*, and D*) were generated using a bi-exponential fitting model implemented in the SYNAPSE VINCENT workstation (FUJIFILM Corporation, Tokyo, Japan), described by the following equation:


$$S(b)=\mathrm{S0}\;[f\cdot \exp(-bD^{*})+(1-f)\cdot \exp(-\mathrm{bD})]$$

where *f* represents the perfusion fraction, *D* the diffusion coefficient, and D* the pseudo-diffusion coefficient.

**Figure 2:**
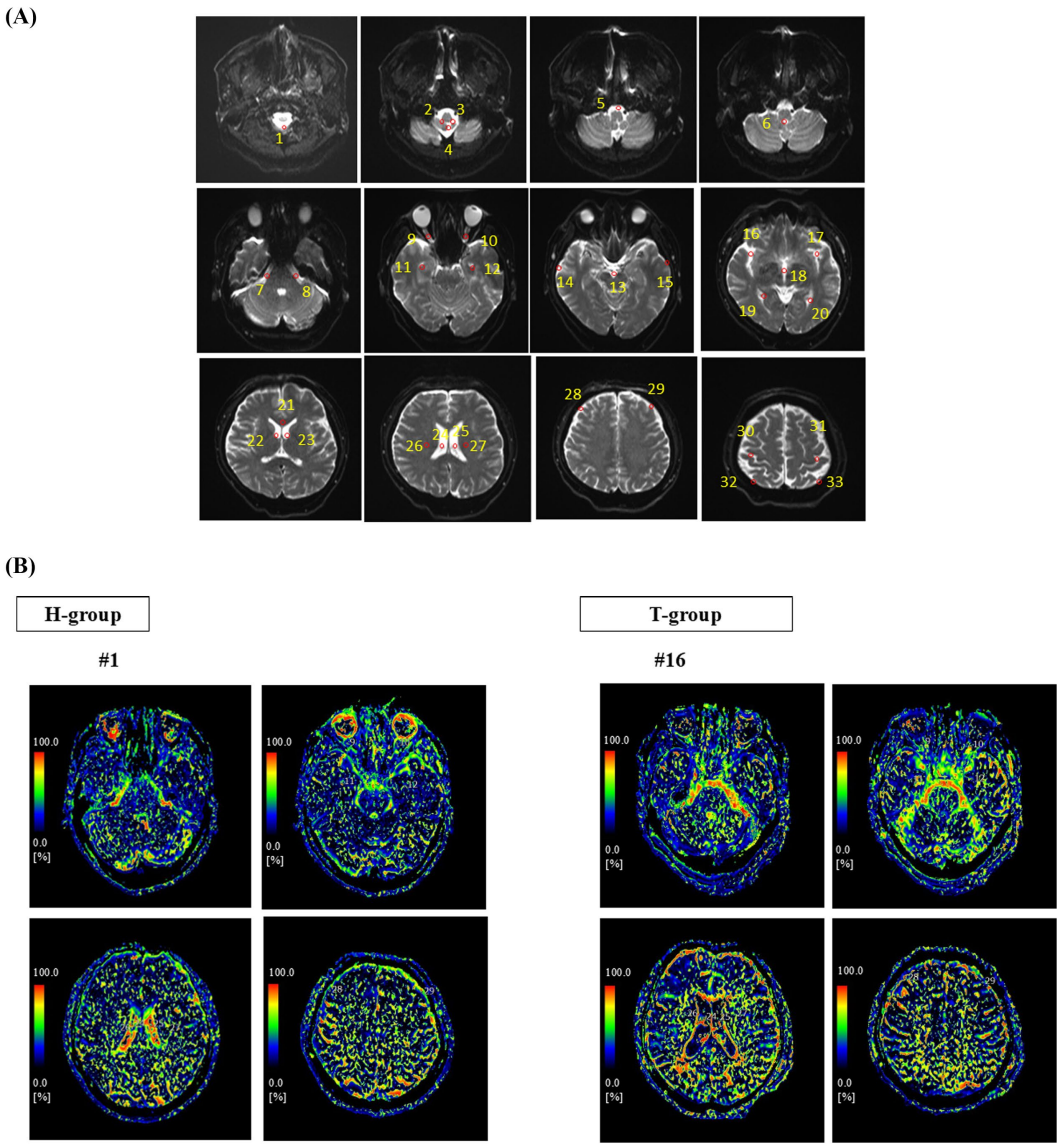
**(A)** Locations of the predefined ROIs used for analysis. Thirty-three ROIs were placed in anatomically defined supratentorial, infratentorial, and parenchymal regions on axial images. ROIs were positioned on *b* = 0 images and applied consistently across all participants. 1. Dorsal caudal medulla oblongata, 2. Right foramen of Luschka, 3. Left foramen of Luschka, 4. Foramen of Magendie, 5. Ventral medulla oblongata, 6. Fourth ventricle, 7. Right cerebellopontine angle, 8. Left cerebellopontine angle, 9. Right optic nerve, 10. Left optic nerve, 11. Right inferior horn of the lateral ventricle, 12. Left inferior horn of the lateral ventricle, 13. Interpeduncular cistern, 14. Right temporal lobe subarachnoid space, 15. Left temporal lobe subarachnoid space, 16. Right Sylvian fissure, 17. Left Sylvian fissure, 18. Third ventricle, 19. Posterior horn of right lateral ventricle, 20. Posterior horn of left lateral ventricle, 21. Splenium of corpus callosum, 22. Right foramen of Monro, 23. Left foramen of Monro, 24. Body of right lateral ventricle, 25. Body of left lateral ventricle, 26. Right corona radiata, 27. Left corona radiata, 28. Right frontal lobe subarachnoid space, 29. Left frontal lobe subarachnoid space, 30. Right central sulcus, 31. Left central sulcus, 32. Right parietal lobe subarachnoid space, and 33. Left parietal lobe subarachnoid space. **(B)** Representative IVIM-derived *f*-maps from participants. The color bar represents the perfusion fraction (*f*), a unitless parameter ranging from 0 to 100% that reflects the fraction of incoherent motion-related signal within a voxel. Warmer colors indicate higher *f*-values, suggesting greater microdynamic motion within the CSF space. These maps illustrate regional variations in CSF motion signals corresponding to the anatomical regions analyzed in panel **(A)**, where the ROI locations are illustrated; representative examples of these regions are also indicated in the present *f*-maps.

IVIM parameters were estimated using a full non-linear fitting procedure based on the Levenberg–Marquardt algorithm implemented in the workstation software. A constraint was applied to the perfusion fraction parameter (0 < *f* < 1), whereas *D* and D* were estimated without explicit positivity constraints. No explicit noise-floor correction was applied during the fitting procedure. Thirty-three regions of interest (ROIs) were predefined *a priori* and manually selected in each participant for analysis according to a previously reported methodology (Yamada et al., 2023). ROI placement was performed by a single rater, a radiological technologist (Y. M.) with 10 years of professional experience. A *b* = 0 image was additionally acquired and used for anatomical reference and ROI placement, with each ROI comprising approximately five contiguous voxels on the axial plane arranged in a cross-shaped configuration (center, superior, inferior, left, and right). To ensure reproducibility, ROIs were symmetrically placed bilaterally at consistent anatomical levels. The selected ROIs included eight infratentorial regions [e.g., fourth ventricle and cerebellopontine angle (CPA)], 20 supratentorial regions (e.g., lateral ventricles and frontal lobe subarachnoid space), and 5 parenchymal regions (e.g., optic nerve, corpus callosum, and corona radiata). Particular care was taken when placing ROIs in CSF spaces to avoid partial-volume contamination from adjacent brain parenchyma or vascular structures. ROI placements were subsequently reviewed by a neurosurgeon (S. W.) to confirm anatomical accuracy and consistency. Figure 2A illustrates the ROI distribution. For each ROI, the IVIM-derived parameters were quantified using the mean *f*-value, which has been shown to effectively evaluate small pulsatile and complex CSF motion within the intracranial CSF spaces (Yamada et al., 2023). In the present study, the IVIM parameter f was used as a surrogate marker of CSF microdynamic motion. The mean *f*-value was designated as the primary endpoint. Representative *f*-maps are presented in Figure 2B. The same predefined ROI template and anatomical levels were applied consistently across all participants to minimize selection bias. For transparency, the mean values of the additional IVIM parameters (*D* and D*) for each ROI are provided in Supplementary Table S1.

### 2.4 Endpoints and statistical analysis

This study compared the CSF motion between the T-group and H-group, with a particular focus on differences in mean *f*-values. For patients with longitudinal MRI data, temporal changes in IVIM parameters were also examined to investigate post-traumatic alterations in CSF dynamics. Continuous variables were expressed as the medians with ranges (minimum to maximum), whereas categorical variables were expressed as counts and percentages. Group comparisons of *f*-values were performed using the unpaired Wilcoxon rank-sum test. Risk ratios (RRs) were estimated based on dichotomized *f*-values, with thresholds determined from the group distributions. Statistical significance was assessed using the unpaired Wilcoxon rank-sum test, and RR estimates were approximated from *χ*^2^ statistics using JMP software version 10.0 (SAS Institute Inc., Cary, NC, USA). All statistical analyses were conducted using JMP version 10, with a *p*-value of <0.050 considered significant. As this study was exploratory and hypothesis generating, ROI-wise analyses were initially summarized using a nominal two-sided p-value of 0.050 without adjustment for multiple comparisons. As a sensitivity analysis, the false discovery rate (FDR) across the 33 ROIs comparisons was controlled using the Benjamini–Hochberg procedure (*q* = 0.05), and adjusted *q*-values were reported. To account for potential confounding by age, a linear regression analysis was performed with *f*-value as the dependent variable and group (TBI = 1; healthy = 0) and age as independent variables. In the T-group, the correlation between f-value and days post-injury was examined using simple regression analysis.

## 3 Results

### 3.1 Clinical baseline characteristics

The T-group was significantly older and had a higher prevalence of antithrombotic therapy, whereas other comorbidities were comparable between the groups. Clinical baseline characteristics are summarized in Table 1A. The median ages were 38 years in the H-group and 61 years in the T-group (*p* < 0.0001). Antithrombotic therapy was administered in five patients in the T-group, whereas none of the healthy controls received such treatment (*p* = 0.04). No significant differences were observed between the group in terms of chronic headache, hypertension, diabetes, dyslipidemia, history of stroke, ischemic heart disease, chronic kidney disease, anemia, smoking, alcohol consumption, or sleep deprivation.

**Table 1:** Baseline characteristics.

(A) Baseline clinical characteristics			
Characteristics	H-group (*N* = 14)	T-group (*N* = 14)	*p*-Value
Sex	Female 9 (64%)	Female 7 (50%)	0.70
Age	Median 38 (27–56) years	Median 61 (41–89) years	<0.0001
Chronic headache	Yes 3 (21%)	0	0.22
Hypertension	Yes 1 (7%)	6 (43%)	0.08
Diabetes mellitus	Yes 1 (7%)	2 (14%)	1.00
Dyslipidemia	Yes 1 (7%)	3 (21%)	0.60
Antithrombotic drug	Yes 0	5 (36%)	0.04
Stroke	Yes 0	3 (21%)	0.22
Ischemic heart disease	Yes 0	1 (7%)	1.00
Chronic kidney disease	Yes 0	1 (7%)	1.00
Anemia	Yes 3 (21%)	4 (29%)	1.00
Smoking habits	Yes 2 (14%)	1 (7%)	1.00
Drinking habits^*^	Yes 1 (7%)	3 (21%)	0.60
Lack of sleep^**^	Yes 6 (43%)	4 (50%)	1.00
^*^One patient in the head trauma group had missing data (NA). ^**^Six patients in the head trauma group had missing data (NA).			

(B) Baseline imaging characteristics			
Characteristics	H-group (*N* = 14)	T-group (*N* = 14)	*p*-Value
Lesion laterality	—	Right: 7, left: 4, both: 2, no: 1	—
Supra/infra-tentorium	—	Supra: 12, both: 1, no: 1	—
Evans index	Median: 0.25 (0.23–0.28)	Median: 0.26 (0.21–0.30)	0.56
DESH	Yes 0	Yes 0	—
DSWMH			0.60^*^
Grade 0	13 (93%)	7 (50%)	
Grade 1	0	1 (7%)	
Grade 2	1 (7%)	3 (21%)	
Grade 3	0	3 (21%)	
Grade 4	0	0	
PVH			1.00^*^
Grade 0	13 (93%)	7 (50%)	
Grade 1	1 (7%)	2 (14%)	
Grade 2	0	4 (29%)	
Grade 3	0	1 (7%)	
Grade 4	0	0	
MRA			0.10^**^
Normal	14 (100%)	9 (64%)	
Irregular wall +	0	1 (7%)	
Stenosis +	0	4 (29%)	
Obstruction +	0	0	
Basal ganglia EPVS			0.48^***^
Grade 0	0	0	
Grade 1	14 (100%)	7 (50%)	
Grade 2	0	5 (36%)	
Grade 3	0	1 (7%)	
Grade 4	0	1 (7%)	
Corticomedullary border EPVS			—
Grade 0	8 (57%)	2 (14%)	
Grade 1	4 (29%)	10 (71%)	
Grade 2	2 (14%)	2 (14%)	
Grade 3	0	0	
Grade 4	0	0	

DESH, disproportionately enlarged subarachnoid space hydrocephalus; DSWMH, deep subcortical white matter hyperintensity; PVH, periventricular hyperintensity; MRA, magnetic resonance angiography; EPVS, enlargement of the perivascular space.

^*^Grade 0 + 1 + 2 or not, ^**^normal + irregular wall or not, ^***^grade 0 + 1 + 2 or not.

### 3.2 Imaging baseline characteristics

Most traumatic lesions were supratentorial, and no significant group differences were observed in brain atrophy, white matter disease, enlarged perivascular space (EPVS) burden, or disproportionately enlarged subarachnoid space hydrocephalus (DESH)-related findings. Imaging baseline characteristics are summarized in Table 1B. The median time from injury to MRI in the T-group was 6 days (interquartile range: 3.75–9.75; range: 2–66). In the T-group, acute lesions were predominantly supratentorial (12/14 patients), with one patient showing both supra- and infra-tentorial involvement and one without detectable lesions. Evans index values were similar between the groups (0.25 in the H-group vs. 0.26 in the T-group; *p* = 0.56) (Evans, 1942), and none of the patients demonstrated DESH features (Hashimoto et al., 2010). Deep subcortical white matter hyperintensity and periventricular hyperintensity grades (Shinohara et al., 2007) were not significantly different between the groups. EPVS grades (Potter et al., 2015) in both the basal ganglia and subcortical white matter were also comparable. Magnetic resonance angiography findings were normal in all controls, whereas only minor irregularities or stenosis were observed in a subset of the T-group (*p* = 0.10).

### 3.3 Acute-phase analysis

**Figure 3:**
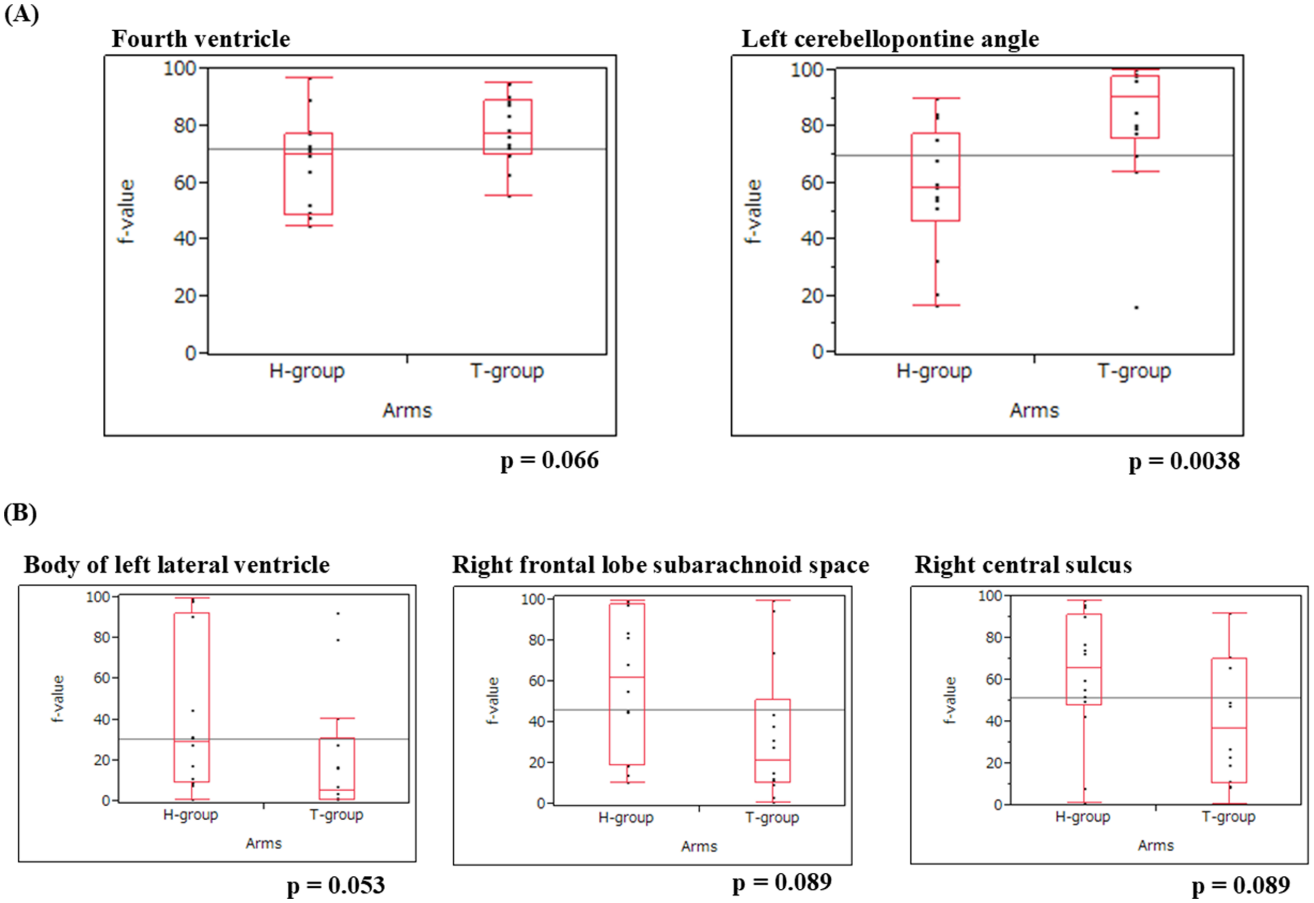
**(A)** Regions of interest with higher cerebrospinal fluid motion in patients with TBI. The fourth ventricle and the left cerebellopontine (CPA) angle exhibited higher mean *f*-values, indicating increased infratentorial cerebrospinal fluid motion in the T-group. Specifically, the fourth ventricle had mean *f*-values of 69.7 (range: 44.9–96.7) and 77.2 (range: 55.4–95.1) (RR^*^: 3.38, *p* = 0.066), whereas the left CPA had *f*-values of 58.0 (range: 16.2–89.8) and 88.1 (range: 15.6–99.9) (RR^*^: 8.38, *p* = 0.0038). ^*^Risk ratios (RRs) were calculated based on *χ*^2^ statistics obtained via Wilcoxon rank-sum tests using JMP software. **(B)** Regions of interest of lower cerebrospinal fluid motion in patients with TBI. The left lateral ventricle, right frontal lobe subarachnoid space,and the right central sulcus exhibited lower mean *f*-values in the T-group. Specifically, the body of the left lateral ventricle had mean *f*-values of 29.0 (range: 0.1–99.4) and 8.6 (range: 0.1–92.2) (RR^*^: 3.74, *p* = 0.053), the right frontal subarachnoid space had mean *f*-values of 61.6 (range: 10.2–99.5) and 28.2 (range: 0.1–99.2) (RR^*^: 2.89, *p* = 0.089), and the right central sulcus had mean *f*-values of 66.5 (range: 0.8–97.9) and 36.7 (range: 0.1–91.5) (RR^*^: 2.89, *p* = 0.089). ^*^Risk ratios (RRs) were calculated based on *χ*^2^ statistics obtained via Wilcoxon rank-sum tests using JMP software.

Acute-phase IVIM analysis demonstrated increased *f*-values in infratentorial regions and a trend toward reduced f-values in selected supratentorial regions (Figure 3, Table 2). In the infratentorial region, the fourth ventricle exhibited mean *f*-values of 69.7 (44.9–96.7) in the H-group and 77.2 (55.4–95.1) in the T-group (RR: 3.38, *p* = 0.066), whereas the left CPA showed values of 58.0 (16.2–89.8) and 88.1 (15.6–99.9), respectively (RR 8.38, *p* = 0.0038), indicating increased CSF motion (Figure 3A). By contrast, supratentorial regions demonstrated a tendency toward lower *f*-values in the T-group, including the body of the left lateral ventricle (29.0 vs. 8.6; *p* = 0.053), the right frontal subarachnoid space (61.6 vs. 28.2; *p* = 0.089), and the right central sulcus (66.5 vs. 36.7; *p* = 0.089) (Figure 3B). After FDR correction across 33 ROIs (*q* = 0.05), no regions remained significant; therefore, these findings should be regarded as exploratory. Regression analysis (Table 3) identified a significant group effect only in the left CPA (*β* = 33.8, *p* = 0.037), with no age effect. Within the T-group, days from injury correlated positively with *f*-values only in the body of the left lateral ventricle (*β* = 1.24, *p* = 0.009).

**Table 2:** Mean *f*-values (%) in ROIs in healthy controls and patients with TBI.

Area		ROI	H-group	T-group	*p*-Value
Infratentorial area	Subarachnoid space	Dorsal caudal portion of medulla oblongata	85.1 (43.9–96.2)	85.8 (46.9–98.7)	0.56
		Rt foramen of Luschka	78.3 (56.1–93.0)	81.8 (65.1–91.6)	0.61
		Lt foramen of Luschka	71.9 (57.1–91.7)	84.1 (53.9–93.7)	0.22
		Foramen of Magendie	73.0 (21.7–94.0)	75.0 (5.3–91.7)	0.66
		Ventral medulla oblongata	76.4 (55.8–90.8)	74.1 (24.6–99.9)	0.88
		Rt CPA	72.6 (13.7–91.4)	73.0 (2.8–99.9)	0.33
		Lt CPA	58.0 (16.2–89.8)	88.1 (15.6–99.9)	0.0038
		Interpeduncular cistern	82.3 (11.7–98.4)	51.1 (6.6–98.7)	0.43
	Intraventricular space	Fourth ventricle	69.7 (44.9–96.7)	77.2 (55.4–95.1)	0.066
Supratentorial area		Third ventricle	83.2 (3.6–99.9)	79.1 (3.1–99.4)	0.36
		Rt inferior horn of lateral ventricle	67.6 (1.2–98.1)	43.4 (1.4–96.8)	0.33
		Lt inferior horn of lateral ventricle	76.9 (19.6–96.3)	74.0 (13.0–99.1)	0.46
		Body of rt. lateral ventricle	11.7 (0.1–95.2)	4.0 (0.1–99.9)	0.29
		Body of lt lateral ventricle	29.0 (0.1–99.4)	8.6 (0.1–92.2)	0.053
		Posterior horn of rt lateral ventricle	91.9 (1.7–98.1)	30.4 (1.4–99.4)	0.38
		Posterior horn of lt lateral ventricle	20.9 (0.1–99.3)	21.3 (0.1–99.1)	0.85
		Rt foramen of Monro	24.0 (0.1–94.5)	42.7 (0.1–99.0)	0.45
		Lt foramen of Monro	20.4 (0.1–93.5)	12.5 (0.1–99.9)	0.80
	Subarachnoid space	Rt Sylvian fissure	56.5 (0.3–97.4)	40.1 (8.6–99.9)	0.65
		Lt Sylvian fissure	34.9 (7.6–95.6)	55.6 (0.7–98.5)	0.61
		Rt temporal lobe subarachnoid space	37.5 (1.8–95.5)	16.3 (1.4–83.7)	0.31
		Lt temporal lobe subarachnoid space	45.2 (21.0–97.7)	41.4 (11.1–97.9)	0.96
		Rt frontal lobe subarachnoid space	61.6 (10.2–99.5)	28.8 (0.1–99.2)	0.089
		Lt frontal lobe subarachnoid space	25.1 (2.0–97.7)	15.2 (2.1–93.1)	0.49
		Rt central sulcus	65.5 (0.8–97.9)	36.7 (0.1–91.5)	0.089
		Lt central sulcus	68.8 (11.3–95.5)	39.0 (0.1–98.8)	0.20
		Rt parietal lobe subarachnoid space	11.8 (0.1–93.8)	24.9 (0.1–98.2)	0.13
		Lt parietal lobe subarachnoid space	14.0 (0.1–99.9)	35.3 (5.4–99.1)	0.23
Nerve/brain		Rt optic nerve	57.4 (0.1–79.3)	32.1 (1.4–87.0)	0.49
		Lt optic nerve	16.6 (6.1–99.9)	10.9 (0.1–88.8)	0.15
		Splenium of corpus callosum	6.6 (2.1–66.8)	5.2 (0.1–83.4)	0.35
		Rt corona radiata	5.9 (0.9–82.8)	30.8 (3.1–77.9)	0.23
		Lt corona radiata	5.3 (0.1–86.9)	7.3 (0.3–78.0)	0.55

ROI, region of interest; Rt, right; Lt, left; CPA, cerebellopontine angle.

**Table 3:** Regression analyses with group and age as independent variables.

ROI	Independent variable	*β*	*p*-Value
Fourth ventricle	Group	3.95	0.69
	Age	0.23	0.32
	Days from injury^a^	−0.20	0.34
Left CPA	Group	33.8	0.037
	Age	−0.25	0.49
	Days from injury^a^	−0.06	0.88
Body of the left lateral ventricle	Group	−23.4	0.33
	Age	0.08	0.89
	Days from injury^a^	1.24	0.009
Right frontal subarachnoid space	Group	−43.2	0.07
	Age	0.48	0.38
	Days from injury^a^	−0.74	0.20
Right central sulcus	Group	−38.2	0.079
	Age	0.51	0.31
	Days from injury^a^	−0.18	0.76

a Days from injury were analyzed only in the TBI group.

ROI, region of interest; CPA, cerebellopontine angle.

### 3.4 Preliminary longitudinal observations in three patients

Follow-up IVIM MRI in three patients revealed divergent temporal trajectories. Supratentorial *f*-values tended to increase over time, whereas infratentorial values either decreased or remained stable. Longitudinal scans were acquired at 40–69 days after injury (patients #9, #13, and #32) (Table 4). In supratentorial regions—including the inferior horns, Sylvian fissures, and frontal subarachnoid space—*f*-values increased in two patients (#9 and #13). By contrast, infratentorial regions (e.g., CPA and ventral medulla), exhibited either decreased or stable f-values. For example, the left CPA in patient #9 decreased from 99.9 to 63.3%, whereas the right CPA in patient #13 decreased from 99.1 to 85.7%. Patient #32, who underwent decompressive surgery, demonstrated pronounced increases in supratentorial *f*-values. These exploratory findings suggest region-dependent and heterogeneous trajectories of CSF microdynamics following mild TBI. The representative *f*-maps (#13 and #32) are shown in Supplementary Figure S1.

**Table 4:** Longitudinal change in *f*-values among patients with TBI.

Area		Patient No.	#9		#13		#32^a^	
			Acute	Sub	Acute	Sub	Acute	Sub
Infratentorial	Subarachnoid space	Dorsal caudal portion of medulla oblongata	63.3	90.3	33.5	95.1	46.9	0.3
		Rt foramen of Luschka	91.6	93.3	77.6	80.3	65.1	79.6
		Lt foramen of Luschka	93.6	79.5	55.9	85.0	74.3	–
		Foramen of Magendie	59.2	86.2	40.7	44.7	–	64.2
		Ventral medulla oblongata	83.3	83.8	88.9	30.6	71.8	67.3
		Rt CPA	93.4	88.7	79.5	63.5	56	70.8
		Lt CPA	97.4	49.7	84.6	80.2	79.3	96.5
		Interpeduncular cistern	50.2	94.6	6.6	96	23.3	14.9
	Intraventricular space	Fourth ventricle	94.3	77.1	70.0	62.2	72.1	88.3
Supratentorial		Third ventricle	67.9	1.7	78.4	23.8	79.1	41.2
		Rt inferior horn of lateral ventricle	85.3	94.7	21.5	96.2	52.9	99.5
		Lt inferior horn of lateral ventricle	95.4	20.2	59.6	91.8	13.9	18.9
		Body of rt lateral ventricle	0.1	3.4	0.1	1.1	1.0	7.9
		Body of lt lateral ventricle	6.8	99.1	0.9	10.3	27.2	8.4
		Posterior horn of rt lateral ventricle	99.4	1.8	99.2	3.2	98.8	4.2
		Posterior horn of lt lateral ventricle	12.1	77.6	7.4	0.7	31.8	99.9
		Rt foramen of Monro	63.1	0.1	0.1	63.5	94.9	15.2
		Lt foramen of Monro	9.3	2.7	8.0	99.5	14.4	6.9
		Rt Sylvian fissure	25.3	82.1	7.6	98.1	56.4	63.8
		Lt Sylvian fissure	98.5	67.4	64.2	5.0	89.0	65.5
	Subarachnoid space	Rt temporal lobe subarachnoid space	83.7	18.5	49.9	3.3	3.0	90.6
		Lt temporal lobe subarachnoid space	72.9	2.7	31.2	47.3	25.1	14.8
		Rt frontal lobe subarachnoid space	30.5	98.2	9.0	93.1	37.4	19.3
		Lt frontal lobe subarachnoid space	5.7	5.3	30.6	22.9	2.1	27.1
		Rt central sulcus	70.3	46.9	22.6	25.9	10.7	25.6
		Lt central sulcus	97.7	9.7	46.3	6.4	58.7	92.5
		Rt parietal lobe subarachnoid space	1.6	13.6	63.1	96	6.3	21.2
		Lt parietal lobe subarachnoid space	99.1	34.3	26.6	93.4	96.3	46.6
Nerve/brain		Rt optic nerve	18.1	28.2	12.4	34.8	20.6	7.2
		Lt optic nerve	0.1	12.3	7.1	48	3.7	77.8
		Splenium of corpus callosum	8.5	5.4	0.2	18.6	6.4	14.5
		Rt corona radiata	3.1	10.8	59.9	62.1	4.1	67.9
		Lt corona radiata	15.5	9.6	3.4	0.3	14.8	1.2

TBI, traumatic brain injury; Sub, subacute; MRI, magnetic resonance imaging.

a This patient underwent decompression surgery between the acute and subacute/chronic-phase MRI scans.

## 4 Discussion

The present study suggests that mild TBI may induce region-specific alterations in CSF microdynamics, characterized by increased motion in infratentorial regions (particularly the fourth ventricle and left CPA) and decreased motion in selected supratentorial regions (including the left lateral ventricle, right frontal subarachnoid space, and right central sulcus). These findings extend previous studies demonstrating that TBI can disrupt CSF pulsation (Kim et al., 2024) and highlight that such alterations are not necessarily uniform across the cranial compartments. By capturing incoherent motion and microperfusion, IVIM MRI facilitated a comprehensive assessment of both supratentorial and infratentorial CSF dynamics, revealing heterogeneous regional responses following TBI.

### 4.1 Regional differences in CSF motion following TBI

#### 4.1.1 Supratentorial regions

Reduced CSF motion in supratentorial regions may result from trauma-related edema, neuroinflammation, and mechanical restriction of CSF pathways. In this study, 13 of 14 patients exhibited supratentorial lesions, suggesting that the local tissue environment may directly contribute to the observed suppression of CSF motion. Dysfunctions of the glymphatic system—potentially mediated by AQP4 depolarization—represent another plausible mechanism. Disruption of AQP4 has been associated with impaired CSF–ISF exchange and cognitive decline (Morita et al., 2023). Furthermore, microglial dysfunction after head trauma may further compromise AQP4 regulation and perivascular clearance, as indicated by evidence from hypoxic microglia models (Xin et al., 2023). The consistently lower *f*-values observed in the left lateral ventricle, right frontal subarachnoid space, and right central sulcus may therefore reflect a combination of localized edema, glymphatic pathway disruption, and reduced perivascular clearance in regions particularly vulnerable to secondary injury mechanisms.

#### 4.1.2 Infratentorial regions

By contrast, increased CSF motion in infratentorial regions, such as the CPA, may reflect compensatory CSF redistribution or altered pressure gradients following TBI. Yamada et al. (2023) described several direct compensatory CSF pathways near the choroid plexus and the foramina of Luschka and Magendie (Yamada and Mase, 2023), which may become more active when supratentorial flow is restricted. Post-traumatic inflammation, tSAH, or transient expansion of subarachnoid spaces may also facilitate increased CSF motion, particularly in the CPA—a region closely associated with brainstem-mediated autonomic regulatory pathways and major CSF outflow structures. The higher *f*-values observed in the left CPA may therefore represent a compensatory enhancement of infratentorial CSF dynamics in response to supratentorial suppression.

#### 4.1.3 CSF dynamics and clinical meanings

These results of region-specific CSF movement provide exploratory insights into the potential regional disruptions in CSF dynamics following TBI and generate hypotheses regarding their clinical implications. The contrasting CSF motion patterns observed between infratentorial and supratentorial regions suggest that post-traumatic changes in neurofluid movement are not uniform throughout the brain but may reflect region-specific responses to injury. Further studies are warranted to elucidate the underlying mechanisms of these alterations and determine their potential contributions to post-traumatic complications, such as secondary hydrocephalus, chronic traumatic encephalopathy, and cognitive dysfunction.

### 4.2 Impact of age and time on CSF dynamics

Age is unlikely to account for the observed group differences. Previous studies reported minimal age-related differences in CSF motion across different age strata (Yamada et al., 2023); in the present study, the significant group effect in the left CPA persisted even after adjusting for age. Temporal factors demonstrated limited influence. Most ROIs did not correlate with days since injury. A notable exception was the body of the left lateral ventricle, where *f*-values exhibited a modest increase over time, suggesting region-specific subacute recovery. Although preliminary, these findings indicate the potential for heterogeneous longitudinal trajectories in CSF microdynamics following TBI.

### 4.3 CSF movement changes across acute- to subacute-phases

Longitudinal IVIM MRI in three patients demonstrated region-specific and divergent trajectories of CSF motion. In supratentorial regions, f-values increased over time in two patients, suggesting recovery from acute suppression, likely driven by edema resolution, resolution of pressure gradients, and attenuation of neuroinflammation. By contrast, infratentorial regions exhibited stable or decreasing *f*-values, consistent with a model in which acute-phase compensatory enhancement gradually normalizes. Surgical decompression in patient #32 produced marked increases in supratentorial *f*-values, highlighting the influence of clinical interventions on longitudinal CSF dynamics. Collectively, these findings indicate that post-TBI CSF motion evolves in a heterogeneous and time-dependent manner, characterized by acute-phase supratentorial suppression and infratentorial enhancement, followed by partial normalization during the subacute period. These patterns are consistent with proposed mechanisms of chronic glymphatic dysfunction after TBI, including AQP4 dysregulation and persistent perivascular clearance impairment.

### 4.4 Comparison with other MRI techniques for CSF dynamics

Several MRI techniques have been employed to evaluate CSF dynamics. Phase-contrast MRI (Enzmann and Pelc, 1991) and four-dimensional flow MRI (Rivera-Rivera et al., 2024) are established methods for directly quantifying bulk CSF flow and pulsatility in major pathways, such as the aqueduct and basal cisterns. Amplified MRI (Holdsworth et al., 2016) has recently been introduced to enhance subtle motion signals, allowing detection of small-amplitude displacements in brain tissue and CSF. Using echo-planar imaging-based functional MRI, Kim et al. (2024) reported a reduction in CSF pulsation after TBI, providing initial evidence that trauma can alter neurofluid dynamics. Although pulsation changes have been demonstrated, the precise regional characteristics and incoherent microfluidic motion remain incompletely understood. By contrast, IVIM MRI (Le Bihan et al., 1986) provides an indirect evaluation of CSF and ISF microdynamics. The *f*-value reflects incoherent motion and microperfusion rather than bulk flow. Although this represents a limitation, IVIM uniquely captures microdynamic changes in cortical and perivascular regions, which are less accessible with conventional flow-based methods. Therefore, IVIM serves as a complementary exploratory approach that expands the MRI repertoire for investigating CSF dynamics. IVIM provides an indirect measure of CSF dynamics, as the f-value reflects incoherent motion and microperfusion rather than true bulk CSF flow. Combining IVIM with additional MRI parameters—such as DWI analysis of the lateral periventricular space (Taoka et al., 2022), diffusion analysis using incrementally strengthened motion-sensitizing gradients (Taoka et al., 2021), and other advanced diffusion metrics—may provide a more comprehensive understanding of abnormalities in CSF circulation.

### 4.5 Limitations and future

This study has some limitations that warrant consideration. First, the sample size was modest, and the study was exploratory in nature. However, the primary aim was hypothesis generation, and the present study is the first to characterize regional CSF microdynamic alterations after TBI using IVIM MRI, providing a foundation for future confirmatory studies. Therefore, future studies incorporating larger cohorts of age-matched healthy controls will be important to better distinguish physiological variation from pathological alterations in CSF microdynamics. Such validation studies will help clarify the robustness and clinical interpretability of IVIM-based CSF motion metrics.

Second, subacute- and chronic-phase data were available for only a subset of patients, limiting the ability to fully characterize the temporal evolution of CSF motion. Although the present analysis focused on the acute phase (median: 6 days post-injury), CSF dynamics are known to evolve over later stages due to neuroinflammatory and compensatory processes. Longitudinal studies with more frequent follow-up imaging are essential to validate these preliminary temporal trends.

Thirdly, the IVIM-derived perfusion fraction (*f*) represents an indirect measure of incoherent motion within a voxel rather than a direct quantification of fluid flow. In the context of CSF dynamics, the *f*-value may reflect microscopic fluid motion or pulsatile displacement within the subarachnoid space rather than bulk CSF flow velocity. In contrast, established techniques such as phase-contrast MRI enable direct measurement of velocity-based CSF flow. Therefore, the present findings should be interpreted as reflecting alterations in microdynamic CSF motion rather than absolute CSF flow. Future studies integrating IVIM MRI with established flow-sensitive techniques, such as phase-contrast MRI or 4D flow MRI, may help further validate the physiological interpretation of IVIM-derived parameters.

Fourthly, methodological considerations relate to the repeatability and reproducibility of IVIM-derived parameters. Previous studies have reported that IVIM parameter estimation may vary depending on acquisition schemes, SNR, and fitting approaches. In particular, the pseudo-diffusion coefficient (D*) and perfusion fraction (*f*) are generally less stable than the diffusion coefficient (D), as they are primarily determined by signal behavior at very low *b*-values and are therefore more sensitive to noise (Andreou et al., 2013; Iima and Le Bihan, 2016). Although acceptable repeatability of IVIM metrics has been demonstrated in brain tissue under controlled imaging conditions, the reproducibility of IVIM-derived measurements in CSF spaces has not yet been fully established. Because CSF spaces provide relatively high signal intensity and minimal microstructural restriction, IVIM fitting in these regions may be relatively stable compared with brain parenchyma; however, this assumption has not yet been systematically validated and requires further investigation in future studies.

Finally, IVIM-derived parameters can be influenced by measurement noise, ROI placement, and partial volume effects. These technical limitations are inherent to diffusion-based imaging but were mitigated through standardized acquisition protocols, consistent ROI definitions, and single-rater evaluation, and subsequent anatomical review by a neurosurgeon.

Despite these constraints, the observed region-specific patterns were coherent and biologically plausible, supporting the internal validity of the findings. Collectively, these limitations indicate that the results should be interpreted with caution; nevertheless, they provide important exploratory insights and generate hypotheses that warrant further investigation in larger, multi-center cohorts using multimodal neurofluid imaging approaches.

## 5 Conclusion

This study provides preliminary insights into region-specific alterations of CSF motion during the acute-phase following mild TBI, with significant changes observed in the left CPA and exploratory trends noted in other supratentorial and infratentorial regions. These findings may suggest that mild TBI-induced alterations in neurofluid dynamics are heterogeneous across the brain, exhibiting distinct regional patterns. These findings should therefore be interpreted as exploratory observations that generate hypotheses regarding regional CSF microdynamic alterations following mild TBI. Given the emerging evidence linking glymphatic dysfunction to neurodegenerative conditions, longitudinal studies that integrate imaging with clinical outcome measures are warranted to determine the clinical relevance of these alterations in post-traumatic neurodegeneration. IVIM MRI may serve as a promising image biomarker for detecting early neurofluid abnormalities in patients with TBI, potentially supporting risk stratification and guiding therapeutic decision-making.

## Data Availability

The original contributions presented in the study are included in the article/Supplementary material, further inquiries can be directed to the corresponding author.

## References

[ref1] Andreou A. Koh D. M. Collins D. J. Blackledge M. Wallace T. Leach M. O. . (2013). Measurement reproducibility of perfusion fraction and pseudodiffusion coefficient derived by intravoxel incoherent motion diffusion-weighted MR imaging in normal liver and metastases. Eur. Radiol. 23, 428–434. doi: 10.1007/s00330-012-2604-1 23052642

[ref2] Daniela D. C. Duarte J. C. González Á. A. O. García J. F. C. (2023). Psychiatric disorders in post-traumatic brain injury patients: a scoping review. Heliyon 19:e12905. doi: 10.1016/j.heliyon.2023.e12905, 36704272 PMC9871203

[ref3] Engelhardt B. Vajkoczy P. Weller R. O. (2017). The movers and shapers in immune privilege of the CNS. Nat. Immunol. 18, 123–131. doi: 10.1038/ni.3666, 28092374

[ref4] Enzmann D. R. Pelc N. J. (1991). Normal flow patterns of intracranial and spinal cerebrospinal fluid defined with phase-contrast cine MR imaging. Radiology 178, 467–474. doi: 10.1148/radiology.178.2.1987610, 1987610

[ref5] Evans W. A. (1942). An encephalographic ratio for estimating ventricular enlargement and cerebral atrophy. Arch. Neurol. Psychiatr. 47, 931–937. doi: 10.1001/archneurpsyc.1942.02290060069004

[ref6] Gao F. Zhao W. Zheng Y. Duan Y. Ji M. Lin G. . (2022). Intravoxel incoherent motion magnetic resonance imaging used in preoperative screening of high-risk patients with Moyamoya disease who may develop postoperative cerebral hyperperfusion syndrome. Front. Neurosci. 16:826021. doi: 10.3389/fnins.2022.826021, 35310102 PMC8924456

[ref7] Hashimoto M. Ishikawa M. Mori E. Kuwana N. (2010). Study of INPH on neurological improvement (SINPHONI). Diagnosis of idiopathic normal-pressure hydrocephalus was supported by an MRI-based prospective cohort study. Cerebrospinal Fluid Res. 27:18. doi: 10.1186/1743-8454-7-18, 21040519 PMC2987762

[ref8] Holdsworth S. J. Rahimi M. S. Ni W. W. Zaharchuk G. Moseley M. E. (2016). Amplified magnetic resonance imaging (aMRI). Magn. Reson. Med. 75, 2245–2254. doi: 10.1002/mrm.26142, 26888418

[ref9] Iima M. Le Bihan D. (2016). Clinical intravoxel incoherent motion and diffusion MR imaging: past, present, and future. Radiology 278, 13–32. doi: 10.1148/radiol.2015150244, 26690990

[ref10] Kim J.-H. Im J.-G. Park S.-H. (2024). Measurement of changes in cerebrospinal fluid pulsation after traumatic brain injury using echo-planar imaging-based functional MRI. NMR Biomed. 37:e5061. doi: 10.1002/nbm.5061, 37839870

[ref11] Kitoka K. Lends A. Kucinskas G. Bula A. L. Krasauskas L. Smirnovas V. . (2024). dGAE(297-391) tau fragment promotes formation of chronic traumatic encephalopathy-like tau filaments. Angew. Chem. Int. Ed. Engl. 63:e202407821. doi: 10.1002/anie.202407821, 39183704 PMC11586700

[ref12] Kwon S. Moreno-Gonzalez I. Taylor-Presse K. Edwards G. III Gamez N. Calderon O. . (2019). Impaired peripheral lymphatic function and cerebrospinal fluid outflow in a mouse model of Alzheimer’s disease. J. Alzheimer's Dis 69, 585–593. doi: 10.3233/JAD-190013, 31104026 PMC7891904

[ref13] Le Bihan D. Breton E. Lallemand D. Grenier P. Cabanis E. Laval-Jeantet M. (1986). MR imaging of intravoxel incoherent motions: application to diffusion and perfusion in neurologic disorders. Radiology 161, 401–407. doi: 10.1148/radiology.161.2.3763909, 3763909

[ref14] Lindfors M. Vehviläinen J. Bendel S. Reinikainen M. Laitio R. Ala-Kokko T. . (2023). Incidence and risk factors of posttraumatic hydrocephalus and its association with outcome following intensive care unit treatment for traumatic brain injury: a multicenter observational study. J. Neurosurg. 139, 1420–1429. doi: 10.3171/2023.2.JNS22728, 37029677

[ref15] Louveau A. Smirnov I. Keyes T. J. Eccles J. D. Rouhani S. J. Peske J. D. . (2015). Structural and functional features of central nervous system lymphatic vessels. Nature 523, 337–341. doi: 10.1038/nature14432, 26030524 PMC4506234

[ref16] Miettinen P. Utz B. Bañuelos-Cabrera I. Golanov E. Lenzner Z. Lara-Valderrábano L. . (2025). Glymphatic system and mild traumatic brain injury: a mini review. Front. Neurosci. 19:1705690. doi: 10.3389/fnins.2025.1705690, 41179995 PMC12572939

[ref17] Miura M. Uchino T. Yamada S. (2022). Description of the latest neurofluid absorption mechanisms with reference to the linkage absorption mechanisms between extravascular fluid pathways and meningeal lymphatic vessels. Auton. Nerv. Syst. 59, 110–124. doi: 10.32272/ans.59.1_110 (English abstract with Japanese article)

[ref18] Møllgård K. Beinlich F. R. M. Kusk P. Miyakoshi L. M. Delle C. Plá V. . (2023). A mesothelium divides the subarachnoid space into functional compartments. Science 379, 84–88. doi: 10.1126/science.adc8810, 36603070

[ref19] Moody J. N. Howard E. Nolan K. E. Prieto S. Logue M. W. Hayes J. P. . (2024). Traumatic brain injury and genetic risk for Alzheimer's disease impact cerebrospinal fluid beta-amyloid levels in Vietnam war veterans. Neurotrauma Rep. 5, 760–769. doi: 10.1089/neur.2024.0048 39184178 PMC11342050

[ref20] Morita Y. Kamagata K. Andica C. Takabayashi K. Kikuta J. Fujita S. . (2023). Glymphatic system impairment in nonathlete older male adults who played contact sports in their youth associated with cognitive decline: a diffusion tensor image analysis along the perivascular space study. Front. Neurol. 14:1100736. doi: 10.3389/fneur.2023.1100736, 36873446 PMC9977161

[ref21] Nedergaard M. (2013). Garbage truck of the brain. Science 340, 1529–1530. doi: 10.1126/science.1240514, 23812703 PMC3749839

[ref22] Plog B. A. Nedergaard M. (2018). The glymphatic system in central nervous system health and disease: past, present, and future. Annu. Rev. Pathol. 13, 379–394. doi: 10.1146/annurev-pathol-051217-111018, 29195051 PMC5803388

[ref23] Potter G. M. Chappell F. M. Morris Z. Wardlaw J. M. (2015). Cerebral perivascular spaces visible on magnetic resonance imaging: development of a qualitative rating scale and its observer reliability. Cerebrovasc. Dis. 39, 224–231. doi: 10.1159/000375153, 25823458 PMC4386144

[ref24] Rivera-Rivera L. A. Vikner T. Eisenmenger L. Johnson S. C. Johnson K. M. (2024). Four-dimensional flow MRI for quantitative assessment of cerebrospinal fluid dynamics: status and opportunities. NMR Biomed. 37:e5082. doi: 10.1002/nbm.5082, 38124351 PMC11162953

[ref25] Shinohara Y. Tohgi H. Hirai S. Terashi A. Fukuuchi Y. Yamaguchi T. . (2007). Effect of the ca antagonist nilvadipine on stroke occurrence or recurrence and extension of asymptomatic cerebral infarction in hypertensive patients with or without history of stroke (PICA study). 1. Design and results at enrollment. Cerebrovasc. Dis. 24, 202–209. doi: 10.1159/000104478, 17596689

[ref26] Taoka T. Ito R. Nakamichi R. Naganawa S. (2022). Diffusion-weighted image analysis along the perivascular space (DWI–ALPS) for evaluating interstitial fluid status: age dependence in normal subjects. Jpn. J. Radiol. 40, 894–902. doi: 10.1007/s11604-022-01275-0, 35474438 PMC9441421

[ref27] Taoka T. Kawai H. Nakane T. Abe T. Nakamichi R. Ito R. . (2021). Diffusion analysis of fluid dynamics with incremental strength of motion proving gradient (DANDYISM) to evaluate cerebrospinal fluid dynamics. Jpn. J. Radiol. 39, 315–323. doi: 10.1007/s11604-020-01075-4, 33389526 PMC8019675

[ref28] Teasdale G. Jennett B. (1974). Assessment of coma and impaired consciousness. A practical scale. Lancet 2, 81–84. doi: 10.1016/s0140-6736(74)91639-0, 4136544

[ref29] Ullah R. Lee E. J. (2023). Advances in amyloid-β clearance in the brain and periphery: implications for neurodegenerative diseases. Exp. Neurobiol. 32, 216–246. doi: 10.5607/en23014, 37749925 PMC10569141

[ref30] von Elm E. Altman D. G. Egger M. Pocock S. J. Gøtzsche P. C. Vandenbroucke J. P. (2007). The strengthening the reporting of observational studies in epidemiology (STROBE) statement: guidelines for reporting observational studies. Lancet 370, 1453–1457. doi: 10.1016/S0140-6736(07)61602-X, 18064739

[ref31] Watanabe S. Shibata Y. Ishikawa E. (2024). A case of idiopathic intracranial hypertension complicated with both infratentorial and supratentorial cortical superficial siderosis: novel imaging findings on intravoxel incoherent motion magnetic resonance imaging offering clues to pathophysiology. Neurol. Int. 16, 701–708. doi: 10.3390/neurolint16040053, 39051214 PMC11270171

[ref32] Wee I. C. Arulsamy A. Corrigan F. Collins-Praino L. (2024). Long-term impact of diffuse traumatic brain injury on neuroinflammation and catecholaminergic signaling: potential relevance for Parkinson’s disease risk. Molecules 29:1470. doi: 10.3390/molecules29071470, 38611750 PMC11013319

[ref33] Wright A. M. Wu Y. C. Feng L. Wen Q. (2024). Diffusion magnetic resonance imaging of cerebrospinal fluid dynamics: current techniques and future advancements. NMR Biomed. 37:e5162. doi: 10.1002/nbm.5162, 38715420 PMC11303114

[ref34] Xin W. Pan Y. Wei W. Tatenhorst L. Graf I. Popa-Wagner A. . (2023). Preconditioned extracellular vesicles from hypoxic microglia reduce poststroke AQP4 depolarization, disturbed cerebrospinal fluid flow, astrogliosis, and neuroinflammation. Theranostics 13, 4197–4216. doi: 10.7150/thno.84059, 37554272 PMC10405850

[ref35] Yamada S. Hiratsuka S. Otani T. Ii S. Wada S. Oshima M. . (2023). Usefulness of intravoxel incoherent motion MRI for visualizing slow cerebrospinal fluid motion. Fluids Barriers CNS 20:16. doi: 10.1186/s12987-023-00415-6, 36899412 PMC9999497

[ref36] Yamada S. Mase M. (2023). Cerebrospinal fluid production and absorption and ventricular enlargement mechanisms in hydrocephalus. Neurol. Med. Chir. 63, 141–151. doi: 10.2176/jns-nmc.2022-0331, 36858632 PMC10166604

