# Supplementary figures and images for: Exploratory assessment of cerebrospinal fluid-related microdynamics after mild traumatic brain injury using intravoxel incoherent motion magnetic resonance imaging
Source: Front Neurosci. 2026 Apr 10;20:1756207. doi: 10.3389/fnins.2026.1756207 (PMC13106457; doi:10.3389/fnins.2026.1756207)

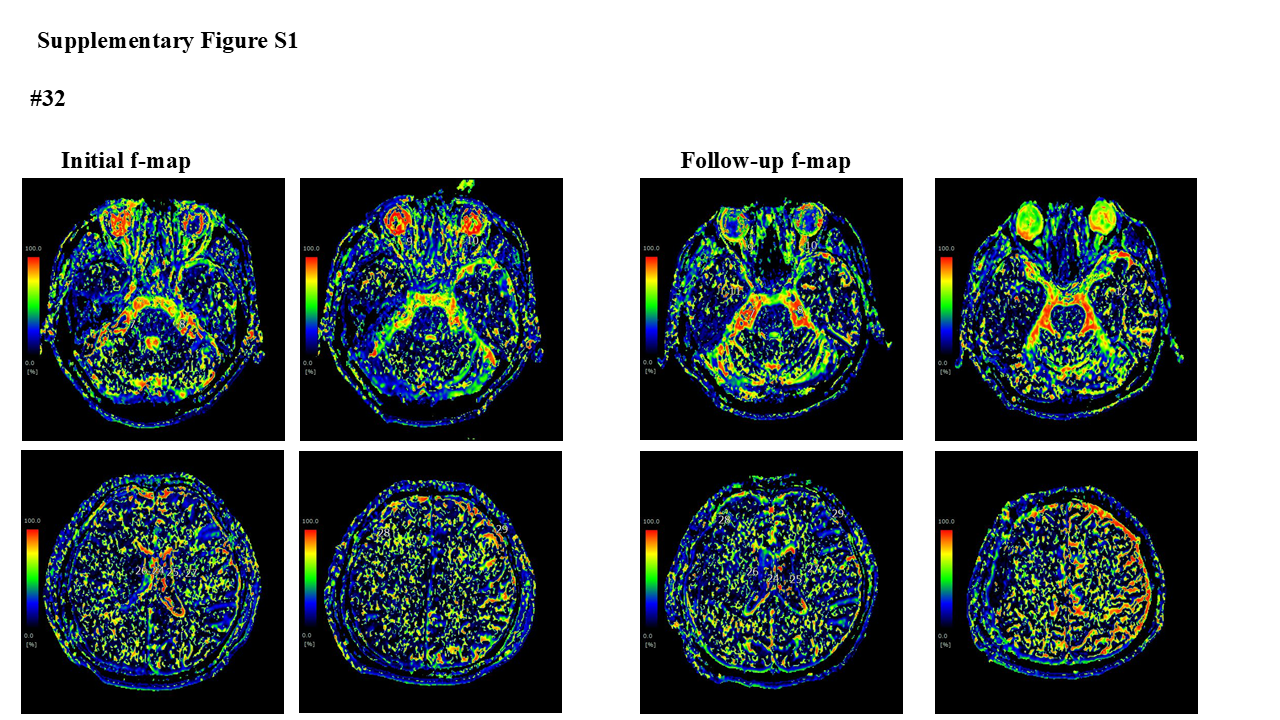

Supplement: Supplementary file 1 [file Image_1.TIF]

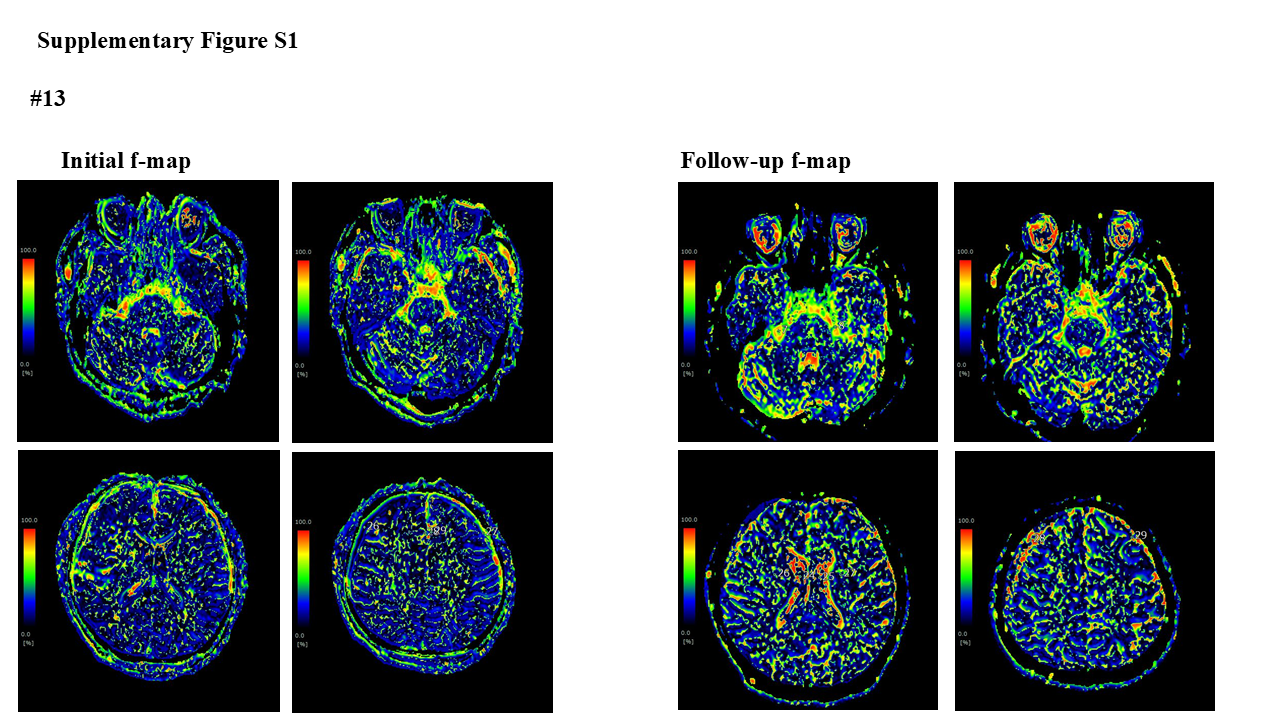

Supplement: Supplementary file 2 [file Image_2.TIF]
