# Supplementary material for: Exploratory assessment of cerebrospinal fluid-related microdynamics after mild traumatic brain injury using intravoxel incoherent motion magnetic resonance imaging
Source: Front Neurosci. 2026 Apr 10;20:1756207. doi: 10.3389/fnins.2026.1756207 (PMC13106457; doi:10.3389/fnins.2026.1756207)
Supplement: Supplementary file 3 [file Table_1.docx]

**Supplementary Table S1-A. Mean D-values in ROIs in healthy controls and patients with TBI**

| **Area** | | **ROI** | **H-group** | **T-group** |
| --- | --- | --- | --- | --- |
| **Infratentorial area** | **Subarachnoid space** | Dorsal caudal portion of medulla oblongata | 1.0 (0.050–4.6) | 1.4 (0.050–5.1) |
|  |  | Rt foramen of Luschka | 2.7 (0.29–18.0) | 1.4 (0.69–16.0) |
|  |  | Lt foramen of Luschka | 2.5 (0.77–18.0) | 1.3 (0.39–11.5) |
|  |  | Foramen of Magendie | 2.9 (0.050–8.5) | 2.2 (0.67–8.6) |
|  |  | Ventral medulla oblongata | 2.3 (0.61–11.7) | 2.2 (0.84–9.8) |
|  |  | Rt CPA | 3.4 (0.080–14.6) | 2.0 (0.050–9.5) |
|  |  | Lt CPA | 4.5 (1.4–12.5) | 1.5 (0.050–6.5) |
|  |  | Interpeduncular cistern | 1.4 (0.050–4.0) | 2.7 (0.050–5.4) |
|  | **Intraventricular space** | Fourth ventricle | 4.3 (0.050–9.7) | 0.94 (0.050–6.2) |
| **Supratentorial area** |  | Third ventricle | 2.4 (0.050–16.6) | 1.6 (0.050–4.0) |
|  |  | Rt inferior horn of lateral ventricle | 1.3 (0.050–3.1) | 1.6 (0.050–3.1) |
|  |  | Lt inferior horn of lateral ventricle | 0.7 (0.050–2.6) | 1.1 (0.050–2.9) |
|  |  | Body of rt lateral ventricle | 2.8 (0.050–3.3) | 3.0 (0.050–3.2) |
|  |  | Body of lt lateral ventricle | 2.4 (0.050–3.3) | 2.9 (1.2–3.2) |
|  |  | Posterior horn of rt lateral ventricle | 0.31 (0.050–3.3) | 2.9 (0.050–3.4) |
|  |  | Posterior horn of lt lateral ventricle | 2.7(0.050–4.5) | 2.9 (0.050–3.6) |
|  |  | Rt foramen of Monro | 2.9 (1.0–7.5) | 2.9 (0.050–3.3) |
|  |  | Lt foramen of Monro | 3.0 (1.0–10.5) | 3.0 (0.050–6.7) |
|  | **Subarachnoid space** | Rt Sylvian fissure | 2.9 (0.050–5.4) | 3.3 (0.050–3.9) |
|  |  | Lt Sylvian fissure | 3.3 (0.45–15.9) | 2.3 (0.050–4.2) |
|  |  | Rt temporal lobe subarachnoid space | 2.2 (0.050–3.1) | 2.2 (0.050–3.0) |
|  |  | Lt temporal lobe subarachnoid space | 1.8 (0.050–2.7) | 1.9 (0.050–3.3) |
|  |  | Rt frontal lobe subarachnoid space | 2.1 (0.050–3.5) | 2.2 (0.050–4.4) |
|  |  | Lt frontal lobe subarachnoid space | 2.4 (0.050–3.2) | 2.6 (0.79–4.4) |
|  |  | Rt central sulcus | 0.76 (0.050–2.9) | 1.3 (0.050–3.1) |
|  |  | Lt central sulcus | 1.3 (0.050–2.9) | 1.3 (0.050–3.2) |
|  |  | Rt parietal lobe subarachnoid space | 2.2 (0.050–3.3) | 1.8 (0.050–3.1) |
|  |  | Lt parietal lobe subarachnoid space | 2.3 (0.050–3.0) | 1.6 (0.050–3.1) |
| **Nerve/brain** | | Rt optic nerve | 0.95 (0.050–2.0) | 1.5 (0.050–2.5) |
|  |  | Lt optic nerve | 1.2 (0.050–1.8) | 1.5 (0.050–2.4) |
|  |  | Splenium of corpus callosum | 0.73 (0.050–0.86) | 0.78 (0.050–0.86) |
|  |  | Rt corona radiata | 0.64 (0.050–0.77) | 0.31 (0.050–0.74) |
|  |  | Lt corona radiata | 0.66 (0.050–0.79) | 0.68 (0.050–0.87) |

ROI: region of interest, Rt: right, Lt: left, CPA: cerebellopontine angle

§D and D* are expressed in ×10⁻³ mm²/s.

**Supplementary Table S1-B. Mean D^*^-values in ROIs in healthy controls and patients with TBI**

| **Area** | | **ROI** | **H-group** | **T-group** |
| --- | --- | --- | --- | --- |
| **Infratentorial area** | **Subarachnoid space** | Dorsal caudal portion of medulla oblongata | 10.9 (3.5–131.3) | 16.9 (3.8–51.6) |
|  |  | Rt foramen of Luschka | 151.6 (42.4–1000.0) | 71.4 (15.4–265.0) |
|  |  | Lt foramen of Luschka | 154.4 (39.2–1000.0) | 126.4 (20.3–1000.0) |
|  |  | Foramen of Magendie | 57.0 (5.1–1000.0) | 44.3 (5.6–1000.0) |
|  |  | Ventral medulla oblongata | 126.1 (26.8–296.9) | 81.5 (4.3–170.6) |
|  |  | Rt CPA | 72.0 (11.1–1000.0) | 22.5 (4.0–1000.0) |
|  |  | Lt CPA | 49.6 (15.5–1000.0) | 14.6 (4.1–1000.0) |
|  |  | Interpeduncular cistern | 9.4 (3.1–1000.0) | 16.1 (3.6–87.8) |
|  | **Intraventricular space** | Fourth ventricle | 141.9 (11.4–1000.0) | 57.3 (4.7–131.6) |
| **Supratentorial area** |  | Third ventricle | 44.0 (3.4–1000.0) | 13.5 (4.0–229.4) |
|  |  | Rt inferior horn of lateral ventricle | 3.9 (1.9–101.6) | 8.0 (1.8–1000.0) |
|  |  | Lt inferior horn of lateral ventricle | 3.9 (2.4–79.1) | 5.0 (2.3–127.2) |
|  |  | Body of rt lateral ventricle | 15.2 (2.6–1000.0) | 3.6 (2.9–1000.0) |
|  |  | Body of lt lateral ventricle | 4.0 (3.2–28.1) | 14.3 (2.9–1000.0) |
|  |  | Posterior horn of rt lateral ventricle | 4.7 (3.3–1000.0) | 3.4 (2.7–1000.0) |
|  |  | Posterior horn of lt lateral ventricle | 8.1 (2.7–94.7) | 7.5 (2.9–33.4) |
|  |  | Rt foramen of Monro | 18.6 (2.8–1000.0) | 5.5 (3.0–1000.0) |
|  |  | Lt foramen of Monro | 6.6 (2.8–1000.0) | 3.2 (2.9–105.7) |
|  | **Subarachnoid space** | Rt Sylvian fissure | 23.5 (4.2–91.0) | 31.2 (2.7–96.1) |
|  |  | Lt Sylvian fissure | 31.7 (10.0–1000.0) | 23.4 (0.34–57.0) |
|  |  | Rt temporal lobe subarachnoid space | 17.1 (1.9–1000.0) | 8.6 (1.8–1000.0) |
|  |  | Lt temporal lobe subarachnoid space | 6.9 (3.2–19.6) | 3.5 (2.6–70.2) |
|  |  | Rt frontal lobe subarachnoid space | 4.3 (2.8–25.9) | 20.5 (3.0–72.3) |
|  |  | Lt frontal lobe subarachnoid space | 3.2 (2.3–31.6) | 9.9 (2.5–122.5) |
|  |  | Rt central sulcus | 3.6 (2.7–7.5) | 7.4 (2.7–29.3) |
|  |  | Lt central sulcus | 3.4 (2.9–9.1) | 5.1 (2.8–36.9) |
|  |  | Rt parietal lobe subarachnoid space | 9.6 (2.5–1000.0) | 6.1 (1.8–143.8) |
|  |  | Lt parietal lobe subarachnoid space | 2.6 (1.7–39.1) | 11.2 (1.7–1000.0) |
| **Nerve/brain** | | Rt optic nerve | 6.7 (1.5–1000.0) | 27.0 (1.4–1000.0) |
|  |  | Lt optic nerve | 6.8 (0.93–1000.0) | 16.5 (1.1–1000.0) |
|  |  | Splenium of corpus callosum | 15.0 (0.72–1000.0) | 4.3 (0.67–1000.0) |
|  |  | Rt corona radiata | 6.9 (0.71–1000.0) | 1.3 (0.56–23.1) |
|  |  | Lt corona radiata | 17.3 (0.61–1000.0) | 5.7 (0.69–1000.0) |

ROI: region of interest, Rt: right, Lt: left, CPA: cerebellopontine angle

§D and D* are expressed in ×10⁻³ mm²/s.
